# 2-Methyl-*N*-*o*-tolyl­benzamide

**DOI:** 10.1107/S1600536809050946

**Published:** 2009-12-04

**Authors:** Aamer Saeed, Rasheed Ahmad Khera, Muhammad Siddiq, Jim Simpson

**Affiliations:** a Department of Chemistry, Quaid-i-Azam University, Islamabad 45320, Pakistan; b Department of Chemistry, University of Otago, PO Box 56, Dunedin, New Zealand

## Abstract

In the title compound, C_15_H_15_NO, the C—N—C(O)—C amide unit is planar (r.m.s. deviation = 0.003 Å) and subtends dihedral angles of 44.71 (5) and 43.33 (5)° with the two *o*-tolyl rings. These aromatic rings are inclined at 4.94 (7)° to one another. The *ortho*-methyl groups of the two tolyl rings are *anti* to one another. In the crystal structure, N—H⋯O hydrogen bonds augmented by C—H⋯π inter­actions link the mol­ecules in a head-to-head fashion into chains along *a*. Independent chains pack in a herringbone pattern along *c*.

## Related literature

For background to our work on benzamide derivatives, see: Saeed *et al.* (2008 ▶). For the 2-methyl-*N*-(3-methyl­phen­yl)benzamide isomer, see: Gowda *et al.* (2008*b* ▶). For other related structures see: Gowda *et al.* (2008*a* ▶,*c* ▶, 2009 ▶).
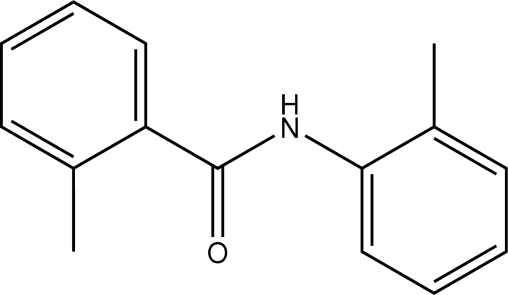


## Experimental

### 

#### Crystal data

C_15_H_15_NO
*M* *_r_* = 225.28
Monoclinic, 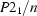

*a* = 4.9340 (4) Å
*b* = 23.639 (2) Å
*c* = 10.0228 (8) Å
β = 91.184 (4)°
*V* = 1168.75 (17) Å^3^
*Z* = 4
Mo *K*α radiation
μ = 0.08 mm^−1^
*T* = 89 K
0.59 × 0.23 × 0.13 mm

#### Data collection

Bruker APEXII CCD area-detector diffractometer
Absorption correction: multi-scan (*SADABS*; Bruker, 2006 ▶) *T*_min_ = 0.762, *T*_max_ = 1.000
19815 measured reflections
3831 independent reflections
3010 reflections with *I* > 2σ(*I*)
*R*_int_ = 0.064

#### Refinement

*R*[*F* ^2^ > 2σ(*F* ^2^)] = 0.055
*wR*(*F* ^2^) = 0.168
*S* = 1.09
3831 reflections
156 parameters
H-atom parameters constrained
Δρ_max_ = 0.80 e Å^−3^
Δρ_min_ = −0.33 e Å^−3^

### 

Data collection: *APEX2* (Bruker, 2006 ▶); cell refinement: *APEX2* and *SAINT* (Bruker, 2006 ▶); data reduction: *SAINT*; program(s) used to solve structure: *SHELXS97* (Sheldrick, 2008 ▶) and *TITAN2000* (Hunter & Simpson, 1999 ▶); program(s) used to refine structure: *SHELXL97* (Sheldrick, 2008 ▶) and *TITAN2000*; molecular graphics: *SHELXTL* (Sheldrick, 2008 ▶) and *Mercury* (Macrae *et al.*, 2006 ▶); software used to prepare material for publication: *SHELXL97*, *enCIFer* (Allen *et al.*, 2004 ▶), *PLATON* (Spek, 2009 ▶) and *publCIF* (Westrip, 2009 ▶).

## Supplementary Material

Crystal structure: contains datablocks global, I. DOI: 10.1107/S1600536809050946/tk2588sup1.cif

Structure factors: contains datablocks I. DOI: 10.1107/S1600536809050946/tk2588Isup2.hkl

Additional supplementary materials:  crystallographic information; 3D view; checkCIF report

## Figures and Tables

**Table 1:** Hydrogen-bond geometry (Å, °)

*D*—H⋯*A*	*D*—H	H⋯*A*	*D*⋯*A*	*D*—H⋯*A*
N1—H1*N*⋯O1^i^	0.88	2.03	2.8891 (13)	166
C31—H31*A*⋯*Cg*1^ii^	0.98	2.76	3.6522 (12)	152
C91—H91*C*⋯*Cg*2^i^	0.98	2.83	3.6999 (12)	148

Symmetry codes: (i) 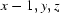; (ii) 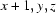. *Cg*1 and *Cg*2 are the centroids of the C2–C7 and C8–C13 benzene rings.

**Fig. 1.**
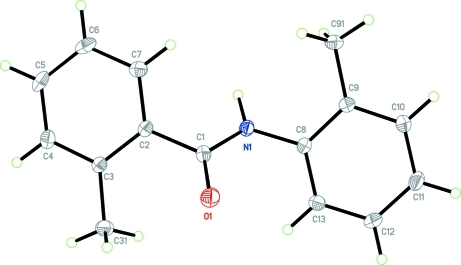
The structure of (I) with displacement ellipsoids for the non-hydrogen atoms drawn at the 50% probability level.

**Fig. 2.**
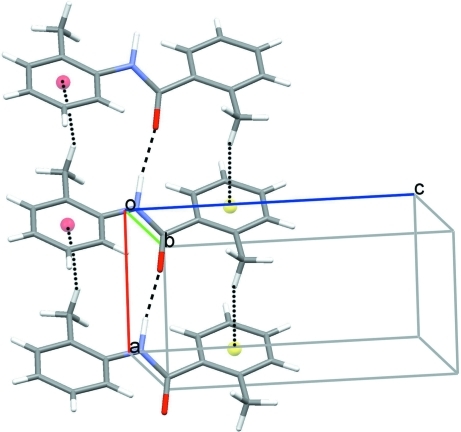
N—H···O hydrogen bonds (dashed lines) and C—H···π interactions in (I) (dotted lines) linking the molecules into chains along a. The coloured spheres represent the ring centroids.

**Fig. 3.**
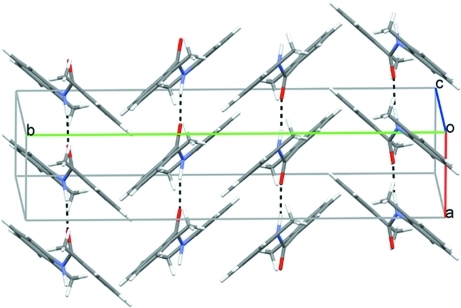
Crystal packing of (I) viewed down the c axis, with hydrogen bonds drawn as dashed lines.

## supplementary crystallographic information

### Comment

The background to our work on benzamide derivatives has been described in a previous paper (Saeed *et al.*, 2008). In the title compound, (I), the C–N–C(O)–C amide unit is planar, r.m.s. deviation 0.003 Å, and subtends dihedral angles of 44.71 (5)° and 43.33 (5)°, respectively, to the two tolyl rings, Fig. 1. These are inclined at 4.94 (7)° to one another giving the overall molecule a stepped structure. The *ortho*-methyl groups of the two tolyl rings are *anti* to one another in contrast to the situation for the isomeric 2-methyl-*N*-(3-methylphenyl)benzamide structure where the methyl substituents are mutually *syn* (Gowda *et al.*, 2008*b*). Bond distances within the molecule are normal and similar to those observed in comparable structures (Gowda *et al.*, 2008*a*,b,c 2009).

In the crystal structure N1—H1N···O1 hydrogen bonds link molecules in a head to head fashion into chains along *b*. This leaves the methyl groups of the two tolyl rings positioned to form C—H···π contacts which reinforce the chain formation, Table 1, Fig. 2. There are no apparent contacts between adjacent chains that generate a herringbone packing motif along *c*, Fig. 3.

### Experimental

*o*-Tolyl chloride (1 mmol) in CHCl_3_ was treated with *o*-toluidine (1 mmol) under a nitrogen atmosphere at reflux for 2 h. Upon cooling, the reaction mixture was diluted with CHCl_3_ and washed consecutively with 1 *M* aq. HCl and saturated aq. NaHCO_3_. The organic layer was dried over anhydrous sodium sulfate and concentrated under reduced pressure. Crystallization of the residue in methanol afforded the title compound (81%) as colourless crystals: Anal. calcd. for C_15_H_15_NO: C, 79.97; H, 6.71; N, 6.22%; found: C, 80.02; H, 6.66; N, 6.36%.

### Refinement

All H-atoms were placed in calculated positions and refined using a riding model with d(N—H) = 0.88 Å, *U*_iso_=1.2*U*_eq_ (N); d(C—H) = 0.95 Å, *U*_iso_=1.2*U*_eq_ (C) for aromatic-H; and 0.98 Å, *U*_iso_ = 1.5*U*_eq_ (C) for CH_3_ H atoms. The final difference Fourier map showed a high peak close to the O1 and H1N atoms.

### Crystal data

**Table:**

C_15_H_15_NO	*F*(000) = 480
*M**_r_* = 225.28	*D*_x_ = 1.280 Mg m^−^^3^
Monoclinic, *P*2_1_/*n*	Mo *K*α radiation, λ = 0.71073 Å
Hall symbol: -P 2yn	Cell parameters from 4591 reflections
*a* = 4.9340 (4) Å	θ = 2.2–31.3°
*b* = 23.639 (2) Å	µ = 0.08 mm^−^^1^
*c* = 10.0228 (8) Å	*T* = 89 K
β = 91.184 (4)°	Triangular, colourless
*V* = 1168.75 (17) Å^3^	0.59 × 0.23 × 0.13 mm
*Z* = 4	

### Data collection

**Table:**

Bruker APEXII CCD area-detector diffractometer	3831 independent reflections
Radiation source: fine-focus sealed tube	3010 reflections with *I* > 2σ(*I*)
graphite	*R*_int_ = 0.064
ω scans	θ_max_ = 31.4°, θ_min_ = 2.2°
Absorption correction: multi-scan (*SADABS*; Bruker, 2006)	*h* = −7→7
*T*_min_ = 0.762, *T*_max_ = 1.000	*k* = −33→22
19815 measured reflections	*l* = −14→14

### Refinement

**Table:**

Refinement on *F*^2^	Primary atom site location: structure-invariant direct methods
Least-squares matrix: full	Secondary atom site location: difference Fourier map
*R*[*F*^2^ > 2σ(*F*^2^)] = 0.055	Hydrogen site location: inferred from neighbouring sites
*wR*(*F*^2^) = 0.168	H-atom parameters constrained
*S* = 1.09	*w* = 1/[σ^2^(*F*_o_^2^) + (0.0962*P*)^2^ + 0.1512*P*] where *P* = (*F*_o_^2^ + 2*F*_c_^2^)/3
3831 reflections	(Δ/σ)_max_ < 0.001
156 parameters	Δρ_max_ = 0.80 e Å^−^^3^
0 restraints	Δρ_min_ = −0.33 e Å^−^^3^

### Special details

**Table:**

Geometry. All e.s.d.'s (except the e.s.d. in the dihedral angle between two l.s. planes) are estimated using the full covariance matrix. The cell e.s.d.'s are taken into account individually in the estimation of e.s.d.'s in distances, angles and torsion angles; correlations between e.s.d.'s in cell parameters are only used when they are defined by crystal symmetry. An approximate (isotropic) treatment of cell e.s.d.'s is used for estimating e.s.d.'s involving l.s. planes.
Refinement. Refinement of *F*^2^ against ALL reflections. The weighted *R*-factor *wR* and goodness of fit *S* are based on *F*^2^, conventional *R*-factors *R* are based on *F*, with *F* set to zero for negative *F*^2^. The threshold expression of *F*^2^ > σ(*F*^2^) is used only for calculating *R*-factors(gt) *etc*. and is not relevant to the choice of reflections for refinement. *R*-factors based on *F*^2^ are statistically about twice as large as those based on *F*, and *R*- factors based on ALL data will be even larger.

### Fractional atomic coordinates and isotropic or equivalent isotropic displacement parameters (Å^2^)

**Table:**

	*x*	*y*	*z*	*U*_iso_*/*U*_eq_	
N1	−0.0409 (2)	0.11397 (4)	0.02480 (10)	0.0144 (2)	
H1N	−0.2109	0.1224	0.0407	0.017*	
C1	0.1521 (2)	0.13178 (5)	0.11331 (11)	0.0135 (2)	
O1	0.39796 (17)	0.12286 (4)	0.09903 (9)	0.0201 (2)	
C2	0.0510 (2)	0.16434 (5)	0.23088 (11)	0.0125 (2)	
C3	0.1575 (2)	0.15349 (5)	0.35932 (12)	0.0132 (2)	
C31	0.3642 (2)	0.10786 (5)	0.38777 (13)	0.0181 (3)	
H31A	0.5405	0.1197	0.3542	0.027*	
H31B	0.3072	0.0728	0.3433	0.027*	
H31C	0.3791	0.1015	0.4843	0.027*	
C4	0.0611 (2)	0.18607 (5)	0.46491 (12)	0.0173 (2)	
H4	0.1289	0.1791	0.5528	0.021*	
C5	−0.1310 (3)	0.22825 (5)	0.44439 (13)	0.0201 (3)	
H5	−0.1926	0.2498	0.5177	0.024*	
C6	−0.2333 (3)	0.23888 (5)	0.31665 (14)	0.0201 (3)	
H6	−0.3640	0.2679	0.3021	0.024*	
C7	−0.1427 (2)	0.20680 (5)	0.21060 (13)	0.0160 (2)	
H7	−0.2131	0.2138	0.1232	0.019*	
C8	0.0152 (2)	0.08234 (5)	−0.09270 (11)	0.0127 (2)	
C9	−0.1221 (2)	0.09560 (5)	−0.21238 (12)	0.0132 (2)	
C91	−0.3265 (2)	0.14277 (5)	−0.22165 (13)	0.0171 (2)	
H91A	−0.3488	0.1547	−0.3149	0.026*	
H91B	−0.2630	0.1749	−0.1676	0.026*	
H91C	−0.5008	0.1295	−0.1885	0.026*	
C10	−0.0626 (2)	0.06309 (5)	−0.32468 (12)	0.0175 (3)	
H10	−0.1525	0.0713	−0.4072	0.021*	
C11	0.1242 (3)	0.01915 (5)	−0.31890 (13)	0.0196 (3)	
H11	0.1618	−0.0021	−0.3968	0.024*	
C12	0.2556 (2)	0.00638 (5)	−0.19899 (12)	0.0175 (2)	
H12	0.3828	−0.0238	−0.1945	0.021*	
C13	0.2009 (2)	0.03771 (5)	−0.08573 (12)	0.0154 (2)	
H13	0.2896	0.0288	−0.0034	0.018*	

### Atomic displacement parameters (Å^2^)

**Table:**

	*U*^11^	*U*^22^	*U*^33^	*U*^12^	*U*^13^	*U*^23^
N1	0.0129 (4)	0.0156 (5)	0.0146 (5)	0.0006 (3)	0.0006 (3)	−0.0028 (3)
C1	0.0161 (5)	0.0119 (5)	0.0125 (5)	−0.0027 (4)	0.0006 (4)	0.0005 (4)
O1	0.0119 (4)	0.0272 (5)	0.0213 (5)	0.0003 (3)	0.0014 (3)	−0.0051 (3)
C2	0.0118 (5)	0.0111 (5)	0.0147 (5)	−0.0022 (3)	0.0016 (4)	−0.0014 (4)
C3	0.0119 (5)	0.0124 (5)	0.0152 (5)	−0.0014 (4)	0.0011 (4)	−0.0016 (4)
C31	0.0164 (6)	0.0188 (5)	0.0192 (6)	0.0022 (4)	−0.0003 (4)	0.0010 (4)
C4	0.0177 (6)	0.0177 (6)	0.0164 (6)	−0.0020 (4)	0.0013 (4)	−0.0045 (4)
C5	0.0211 (6)	0.0157 (5)	0.0238 (6)	−0.0007 (4)	0.0061 (5)	−0.0082 (4)
C6	0.0173 (6)	0.0124 (5)	0.0307 (7)	0.0027 (4)	0.0027 (5)	−0.0023 (4)
C7	0.0141 (5)	0.0132 (5)	0.0208 (6)	−0.0011 (4)	−0.0014 (4)	0.0010 (4)
C8	0.0127 (5)	0.0123 (5)	0.0131 (5)	−0.0015 (4)	0.0019 (4)	−0.0005 (4)
C9	0.0114 (5)	0.0128 (5)	0.0153 (5)	−0.0007 (4)	0.0008 (4)	0.0001 (4)
C91	0.0150 (5)	0.0165 (5)	0.0197 (6)	0.0030 (4)	0.0002 (4)	0.0017 (4)
C10	0.0188 (6)	0.0194 (6)	0.0143 (5)	0.0016 (4)	−0.0021 (4)	−0.0022 (4)
C11	0.0227 (6)	0.0186 (6)	0.0178 (6)	0.0019 (4)	0.0020 (5)	−0.0059 (4)
C12	0.0166 (6)	0.0148 (5)	0.0211 (6)	0.0035 (4)	0.0014 (4)	−0.0012 (4)
C13	0.0147 (5)	0.0152 (5)	0.0162 (6)	0.0008 (4)	−0.0011 (4)	0.0005 (4)

### Geometric parameters (Å, °)

**Table:**

N1—C1	1.3553 (15)	C6—H6	0.9500
N1—C8	1.4270 (14)	C7—H7	0.9500
N1—H1N	0.8800	C8—C13	1.3980 (16)
C1—O1	1.2423 (14)	C8—C9	1.4007 (16)
C1—C2	1.5013 (16)	C9—C10	1.3989 (16)
C2—C7	1.3979 (16)	C9—C91	1.5054 (16)
C2—C3	1.4040 (16)	C91—H91A	0.9800
C3—C4	1.4000 (16)	C91—H91B	0.9800
C3—C31	1.5074 (16)	C91—H91C	0.9800
C31—H31A	0.9800	C10—C11	1.3891 (17)
C31—H31B	0.9800	C10—H10	0.9500
C31—H31C	0.9800	C11—C12	1.3869 (18)
C4—C5	1.3881 (17)	C11—H11	0.9500
C4—H4	0.9500	C12—C13	1.3867 (17)
C5—C6	1.3893 (19)	C12—H12	0.9500
C5—H5	0.9500	C13—H13	0.9500
C6—C7	1.3872 (17)		
			
C1—N1—C8	123.87 (10)	C6—C7—C2	120.75 (11)
C1—N1—H1N	118.1	C6—C7—H7	119.6
C8—N1—H1N	118.1	C2—C7—H7	119.6
O1—C1—N1	123.13 (11)	C13—C8—C9	121.08 (10)
O1—C1—C2	121.24 (10)	C13—C8—N1	119.50 (10)
N1—C1—C2	115.62 (10)	C9—C8—N1	119.40 (10)
C7—C2—C3	120.41 (10)	C10—C9—C8	117.42 (10)
C7—C2—C1	119.42 (10)	C10—C9—C91	120.57 (10)
C3—C2—C1	120.12 (10)	C8—C9—C91	122.01 (10)
C4—C3—C2	117.78 (10)	C9—C91—H91A	109.5
C4—C3—C31	119.32 (11)	C9—C91—H91B	109.5
C2—C3—C31	122.89 (10)	H91A—C91—H91B	109.5
C3—C31—H31A	109.5	C9—C91—H91C	109.5
C3—C31—H31B	109.5	H91A—C91—H91C	109.5
H31A—C31—H31B	109.5	H91B—C91—H91C	109.5
C3—C31—H31C	109.5	C11—C10—C9	121.81 (11)
H31A—C31—H31C	109.5	C11—C10—H10	119.1
H31B—C31—H31C	109.5	C9—C10—H10	119.1
C5—C4—C3	121.65 (12)	C12—C11—C10	119.79 (11)
C5—C4—H4	119.2	C12—C11—H11	120.1
C3—C4—H4	119.2	C10—C11—H11	120.1
C4—C5—C6	120.02 (11)	C11—C12—C13	119.84 (11)
C4—C5—H5	120.0	C11—C12—H12	120.1
C6—C5—H5	120.0	C13—C12—H12	120.1
C7—C6—C5	119.38 (11)	C12—C13—C8	120.05 (11)
C7—C6—H6	120.3	C12—C13—H13	120.0
C5—C6—H6	120.3	C8—C13—H13	120.0

### Hydrogen-bond geometry (Å, °)

**Table:**

*D*—H···*A*	*D*—H	H···*A*	*D*···*A*	*D*—H···*A*
N1—H1N···O1^i^	0.88	2.03	2.8891 (13)	166
C31—H31A···Cg1^ii^	0.98	2.76	3.6522 (12)	152
C91—H91C···Cg2^i^	0.98	2.83	3.6999 (12)	148

Symmetry codes: (i) *x*−1, *y*, *z*; (ii) *x*+1, *y*, *z*.
